# Frequency of tremor in people with multiple sclerosis: A systematic review and meta-analysis

**DOI:** 10.1016/j.prdoa.2025.100315

**Published:** 2025-03-14

**Authors:** Saeed Vaheb, Danial Dehghani Firouzabadi, Hamed Ghoshouni, Mohammad Yazdan Panah, Vahid Shaygannejad, Omid Mirmosayyeb

**Affiliations:** a Isfahan Neurosciences Research Center, Isfahan University of Medical Sciences, Isfahan, Iran; b Student Research Committee, Shahid Sadoughi University of Medical Sciences, Yazd, Iran; c Student Research Committee, Shahrekord University of Medical Sciences, Shahrekord, Iran; d Department of Neurology, Isfahan University of Medical Sciences, Isfahan, Iran

**Keywords:** Multiple sclerosis, Tremor, Frequency

## Abstract

**Background:**

Multiple sclerosis (MS) is a chronic neurodegenerative disorder causing various symptoms, including tremors, which significantly affect the quality of life and disability in people with MS (PwMS). Previous studies report a wide range of tremor frequency in PwMS, necessitating a comprehensive review for reliable estimates.

**Objectives:**

This review aimed to elucidate the frequency rate of tremor among PwMS.

**Method:**

A systematic search was conducted in PubMed/MEDLINE, Embase, Scopus, and Web of Science up to April 27, 2024, to identify studies evaluating various types of tremors in PwMS. The Meta-proportion method implemented in R software version 4.0.0, utilizing a random-effects model, was employed to estimate the pooled frequency rates of tremor, with its 95% confidence interval (CI), among PwMS.

**Results:**

From 3780 studies, 14 studies encompassing 17,458 PwMS (71.5 % female) were included. The mean age was 46.4 years, with a disease duration of 9.3 years and an Expanded Disability Status Scale (EDSS) score of 3.4. The pooled frequency of tremor was 33.32 % (95 % CI: 23.47 % to 44.88 %; I^2^ = 98 %; *p*-heterogeneity < 0.01). Subgroup analysis by sample size revealed that the pooled frequency of tremor in PwMS was significantly lower (*p*-value < 0.01) in studies with over 200 participants (22.46, 95 % CI: 15.69 % to 31.08 %, I^2^ = 99 %; *p*-heterogeneity < 0.01) compared to those with fewer than 200 participants (47.65, 95 % CI: 31.97 % to 63.81 %, I^2^ = 91 %; *p*-heterogeneity < 0.01)

**Conclusion:**

Tremor is a prevalent complaint in PwMS. These findings highlight the necessity for targeted supportive, therapeutic, and rehabilitative interventions to effectively address this prevalent issue in PwMS.

## 1 Introduction

Multiple sclerosis (MS) is a chronic inflammatory dysfunction of the central nervous system (CNS) that can cause a wide variety of symptoms, depending on the specific location of the brain or spinal cord affected [1]. In people with MS (PwMS), symptoms such as sensory and motor disturbances, imbalance, diplopia, and tremors are observed [2]. Following the autoimmune inflammation in the CNS, lesions develop that can destroy astrocytes and, finally, lead to neurodegeneration [3]. Depending on the location and extent of these lesions, different symptoms occur in PwMS, which can lead to different levels of disability [4].

Tremor is typically defined as a rhythmic, oscillatory, sinusoidal movement about a joint produced by contractions of reciprocally innervated antagonist muscles [5], can appear in different parts of the body, such as the upper and lower limbs, head, face, vocal cords, and trunk [5]. Essential tremor can be observed in different conditions, such as Parkinson's disease, hyperthyroidism, and MS [6]. Tremors in PwMS, depending on the location of CNS lesions, can be seen in different types [7]. 1: Action tremor: This category includes intention tremor and postural tremor. Intention tremor typically emerges when an individual intends to perform a voluntary action and is more common in patients with lesions in the cerebellum and related pathways [8]. Postural tremor often results from lesions in the pathways of cerebellar projections to the thalamocortical tract or pons and appears after maintaining a specific position in patients [9].

2: Resting tremor: Observed in the resting state, this type of tremor is often associated with lesions in the midbrain. Although more common in patients with Parkinson’s disease, it can also be a symptom in PwMS with brainstem lesions [10].

Tremor, as a symptom characterized by a notable resistance to pharmacological intervention [7], [11], is closely associated with a decline in quality of life and an increase in disability among PwMS [12]. This condition poses considerable challenges for patients and healthcare providers [12]. The initial step in exploring tremors in PwMS involves assessing their frequency; however, existing literature has reported a wide variability in tremor frequency rates, ranging from 14 % to 68 % [13], [14]. Consequently, there is a pressing need for a comprehensive review to establish a more accurate frequency rate of tremors in PwMS. This review aimed to quantify the frequency of tremors in PwMS, thereby providing reliable data for clinicians and the scientific community.

## 2 Method

### 2.1 Study design

This systematic review and *meta*-analysis were conducted following the Preferred Reporting Items for Systematic Reviews and Meta-Analyses (PRISMA) guidelines [15].

### 2.2 Search strategy

To perform a comprehensive search, we searched PubMed/MEDLINE, Embase, Scopus, and Web of Science to find all relevant studies up to April 27, 2024. The search methodology employed any related MeSH terms of multiple sclerosis and tremor and was then customized for the respective databases. Additional studies were identified manually by reviewing reference lists from relevant articles. Further details of the search strategy are available in Supplementary Material 1.

### 2.3 Study selection

Two review authors (DD and SV) independently screened titles and abstracts using EndNote software (EndNote X21, Thomson Reuters, New York). If an abstract seemed relevant to the review question, the full-text article was obtained for further assessment. In cases of disagreement between the two reviewers, a third reviewer (OM) was consulted to resolve the issue.

### 2.4 Inclusion and exclusion criteria

Studies were included based on the following criteria:

Written in English,
Peer-reviewed,
Cohort and cross-sectional design,
Conducted on people with a confirmed diagnosis of MS according to the accepted diagnostic criteria,
Investigating any type of tremor in PwMS.
Only studies that diagnosed MS based on the McDonald criteria or derived data from reputable hospital-based or registry sources were included.

Studies were excluded based on the following criteria:

Non-English articles,
Case reports and case series,
Animal and in-vitro studies,
Studies with insufficient or ambiguous information,
Conference abstracts, editorials, letters, protocols, and review articles
Studies that identified tremors using telephone-based methods or other methods that did not involve a physician's confirmed diagnosis.
Studies focusing on rhythmic movement disorders other than tremor (e.g., myorhythmia, palatal and segmental myoclonus)

### 2.5 Data extraction

Two reviewers (MYP and DD) extracted the desired data, including details such as the first author, publication year, country of origin, study design, sample size and demographic characteristics of PwMS, Expanded Disability Status Scale (EDSS) scores, disease duration, sample size of pwMS with tremor, type of tremor, site of tremor, and the diagnostic methods for tremor used in the included studies.

### 2.6 Risk of bias assessment

The risk of bias (ROB) assessment was conducted using the Newcastle–Ottawa Scale (NOS) [16]. The NOS checklist evaluated the quality of our studies based on three dimensions: selection, comparability, and outcome. Two reviewers (HG and MYP) performed the quality assessment, and any disagreements were resolved by a third researcher (OM).

### 2.7 Data analysis

The *meta*-analysis used R version 4.4.0 and the “meta” package. Heterogeneity between studies was assessed using the inconsistency index (I^2^) and the p-value for the Q-Cochrane test. A significant level of heterogeneity was shown when the I^2^ value was more than 50 %. The subgroup analysis and *meta*-regression were performed to identify the source of heterogeneity within the studies. The random effects model using the inverse variance method was applied due to high statistical and methodological heterogeneity among the included studies. The funnel plot, Egger's, and Begg's tests were used to assess publication bias. A P-value less than 0.05 was considered significant in all analyses.

## 3 Results

### 3.1 Literature search

**Fig. 1:**
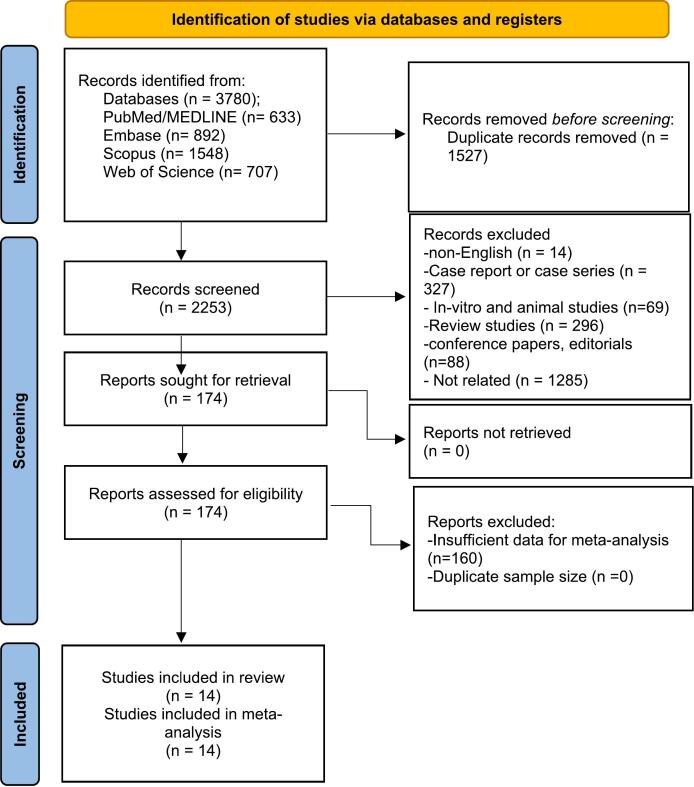
PRISMA 2020 flow diagram of the study process.

In an initial literature search, 3780 studies were identified. After removing 1527 duplicates by EndNote, 2253 articles remained for the title and abstract screening. From this screening, 174 studies moved forward to the full-text review. However, 160 of these studies were excluded due to insufficient data. Finally, 14 articles were included in the qualitative and quantitative syntheses. Additional details are presented in the PRISMA flowchart in Fig. 1.

### 3.2 Study characteristics

The included studies encompassed 17,458 PwMS (71.5 % female). The mean (SD) age was 46.4 (7.8) years, with a mean (SD) disease duration of 9.3 (2.6) years and a mean (SD) EDSS score of 3.4 (1.5). 74.4 % of PwMS had tremors in upper limbs, 7.7 % in lower limbs and head, 3.7 % in trunk, and 1.3 % in vocal cords. Additionally, among the included studies, 45.6 % of tremors were classified as intention tremors, 37 % as action tremors, and 17.3 % as resting tremors. The included studies originated from eight countries: four from the USA [17], [18], [19], [20], three from the UK [21], [22], [23], two from Iran [24], [25], and one each from France [26], Switzerland [27], Egypt [28], and Brazil [29]. Comprehensive details of included studies are outlined in Table 1.

**Table 1:** Fundamental characteristics of included studies.

First Author	Country Year	Study Design	Sample Size F:M Age; years: mean (SD)	EDSS	Disease Duration; years: mean (SD)	MS Subtype	Diagnostic Criteria of Tremor	Number of PwMS with tremor	Tremor Severity	Tremor Site	Tremor Type	QA
HG Briceño [43]	Spain 2024	Cross-sectional	73 39:34 NR	6.5 (1.5)	NR	RRMS: 12 SPMS: 47 PPMS: 14	Clinical	28	NR	NR	NR	7
A Shalash [28]	Egypt 2023	Cross-sectional	250 164:86 33.1 (8.3)	2.5 (0.9)	4.7 (4.2)	RRMS: 250	Clinical, Hospital based registry	36	Mild: 26 Moderate: 10 Severe: 0	NR	NR	9
M Salari [25]	Iran 2018	Cross-sectional	164 132:32 36.3 (9.3)	2.7 (1.8)	NR	RRMS: 104 SPMS: 48 PPMS: 12	Clinical	113	NR	Upper limbs: 113	Resting: 50 Action: 107 Intention: 72	7
L Barin [27]	Switzerland 2018	Cross-sectional	855 622:233 48 (38–57)*	NR	9 (3.5-16)*	RRMS: 611 SPMS: 147 PPMS: 97	Clinical, Hospital based registry	197	NR	NR	NR	10
CC da Silva [29]	Brazil 2018	Cross-sectional	208 NR NR	NR	NR	NR	Clinical, Hospital based registry	26	NR	Upper limbs: 20 Head: 7 Trunk: 4 Vocal cords: 4 Lower limbs: 1	Intention: 19	7
HJ Gross [19]	USA 2017	Cross-sectional	563 387:176 NR	NR	NR	RRMS: 458 SPMS: 105	Clinical, Hospital based registry	97	NR	NR	NR	7
JR Rinker [18]	USA 2015	Cross-sectional	13873 NR NR	NR	NR	NR	Clinical, Hospital based registry	6340	Severe: 805 NR: 5535	NR	NR	10
SS Ayache [26]	France 2015	Cross-sectional	32 23:9 NR	NR	NR	RRMS: 18 SPMS:15	Clinical	18	NR	NR	NR	5
GD Cochrane [20]	USA 2015	Cross-sectional	70 NR NR	NR	NR	NR	Clinical	26	NR	NR	Intention: 26	6
M Rahmanian [24]	Iran 2014	Cross-sectional	81 55:26 31.2 (9.1)	4 NR	8.55 NR	RRMS: 68 SPMS: 9 PPMS: 4	Clinical	12	NR	NR	NR	7
JP Zajicek [23]	UK 2010	Cross-sectional	967 725:242 51.6 (11.5)	NR	9.9 (2.9-15.9)*	RRMS: 347 SPMS: 184 PPMS: 202 NR: 29	Clinical, Hospital based registry	251	NR	NR	NR	7
SJ Pittock [17]	USA 2004	Cross-sectional	200 140:60 NR	3 (0-9.5)*	NR	RRMS: 129 SPMS: 60 PPMS: 11	Clinical, Hospital based registry	51	NR	Upper limbs: 47 Head: 7 Lower limbs: 12	NR	5
SH Alusi [22]	UK 2001	Cross-sectional	100 65:35 47.2 (10.3)	6 (0-9)*	18.8 (11.2)	RRMS: 15 SPMS: 63 PPMS: 22	Clinical	58	Detectable: 27 Mild: 16 Severe: 15	Upper limbs: 56 Lower limbs: 10 Head: 9 Trunk: 7	NR	5
X Liu [21]	UK 1997	Cross-sectional	22 14:8 38 (21–51)*	NR	NR	RRMS: 14 SPMS: 8	Clinical	15	Severe: 5 NR: 10	NR	Intention: 15	3

*Median (range), **Median (IQR), ^m^Months, Mean (Range)^α^. Median^R^. EDSS: Expanded Disability Status Scale, NR: not reported, PwMS: people with multiple sclerosis, PPMS: primary progressive MS, PRMS: progressive relapsing MS, QA: Quality Assessment, RRMS: relapsing remitting MS, SPMS: secondary progressive MS.

### 3.3 Overall frequency of tremor in people with multiple sclerosis

**Fig. 2:**
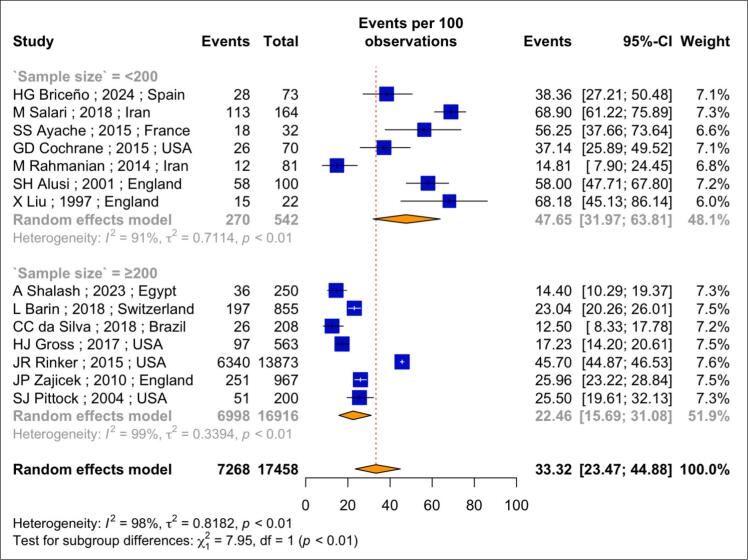
Forest plot of the *meta*-analysis of pooled prevalence of tremor in people with multiple sclerosis.

Based on data from 17,458 PwMS and analyzed using a random effects model, the pooled frequency of tremor was found to be 33.32 % (95 % CI: 23.47 % to 44.88 %; I^2^ = 98 %; *p*-heterogeneity < 0.01) (Fig. 2). Among four studies that assessed tremor severity, 44.6 % of reported cases were mild, 27.7 % were moderate, and 12.8 % were severe.

### 3.4 Subgroup analysis and *meta*-regression

Due to the high heterogeneity observed among the included studies, a subgroup analysis was performed to identify its source. The subgroup analysis based on sample size showed that the pooled frequency of tremor was 47.65 % (95 % CI: 31.97 %-63.81 %; I^2^ = 91.0 %; *p*-heterogeneity < 0.01) in seven studies with sample size fewer than 200 PwMS. In contrast, the frequency was 22.46 % (95 % CI: 15.69 %-31.08 %; I^2^ = 98.0 %; *p*-heterogeneity < 0.01) in studies with a sample size of 200 or more PwMS. This difference was statistically significant (*p*-value < 0.01).

Furthermore, a meta-regression was conducted to analyze the potential sources of heterogeneity. The results showed that neither the publication year (*p*-value = 0.12) nor the sample size (*p*-value = 0.64) significantly affected heterogeneity.

### 3.5 Publication bias

**Fig. 3:**
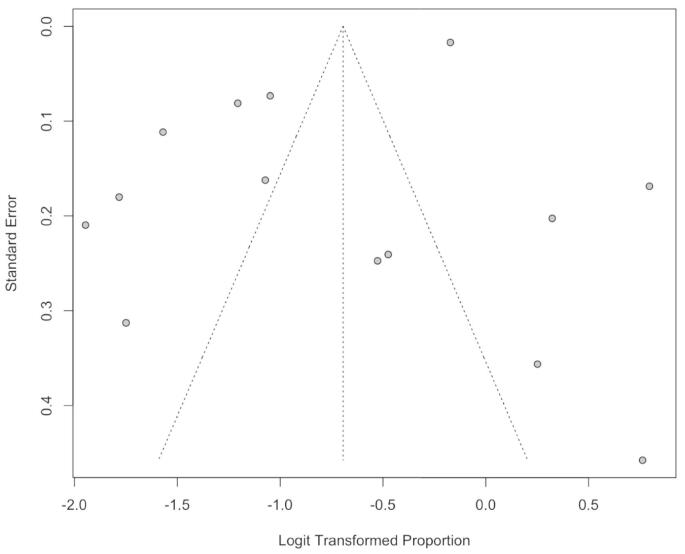
Funnel plot of the *meta*-analysis of pooled prevalence of tremor in people with multiple sclerosis.

Publication bias was assessed using a funnel plot and Egger's and Begg's tests. The funnel plot did not suggest significant asymmetry, indicating a low risk of publication bias. Additionally, Egger's test (*p*-value = 0.07) and Begg's test (*p*-value = 0.25) demonstrated no significant publication bias (Fig. 3).

## 4 Discussion

Based on the findings from this systematic review and *meta*-analysis, which analyzed data from 14 studies involving 17,458 PwMS across nine countries, the estimated frequency of tremor among PwMS was 33.32 %. Furthermore, in the subgroup analysis of studies with a sample size of more than 200 participants, the frequency of tremor was 22.46 %.

The studies reviewed in this *meta*-analysis reported a wide range of tremor frequency reports. The dissimilarity of these reports is likely due to various factors, such as the stage of the disease, the location of MRI lesions, disease duration, and EDSS [14], [20].

Due to the significant heterogeneity of study results and the influence of various parameters on tremor frequency, this study conducted a subgroup analysis based on the sample sizes of included studies. This approach enables us to generalize findings from studies with larger populations, particularly epidemiological studies, to the entire population of PwMS. These results indicated that the frequency of tremors in PwMS, based on studies with more than 200 patients [14], [17], [18], [27], [29], [30], [31], is 22.46 %. This likely reflects a lower risk of selection bias, enhancing the validity of the findings. Consequently, it can be concluded that tremors occur in approximately one-quarter to one-third of PwMS.

Tremor, one of the main symptoms in MS patients, can significantly decrease the quality of life of PwMS [29]. This reduction in the quality of life is usually caused by motor dysfunctions, social stigma, inability to find a job, and disturbance in social activities, which can eventually be associated with depression [18]. In this regard, A. Shalash et al. found a significant difference in the severity of tremors between PwMS with and without depression. The observed association between depression and tremor severity may be explained by the increased perception of disability in individuals with tremor, potentially exacerbated by underlying depressive symptoms [14].

One of the main factors related to the occurrence and severity of tremors is the MS phase. For example, A. Shalash et al. demonstrated that the risk of incidence or exacerbation of tremor in people with relapsing-remitting MS (PwRRMS) often increases during relapse and decreases dramatically in the remission phase [14]. Regardless, this association between the occurrence of tremors and relapse in people with progressive MS (PwPMS) becomes meaningless due to the destruction of cerebellar pathways and progressive neurodegeneration, resulting in patients often experiencing tremors permanently [22], [32]. This difference in pathogenesis between MS subtypes is probably one of the leading causes of prevalence heterogeneity reported in studies. Furthermore, previous research has shown that the severity of tremors reported in PwRRMS is frequently mild to moderate [17], [25], making it challenging for neurologists to diagnose this type of tremor and leading to false negative results in studies, adding to the heterogeneity of prevalence findings.

Prior MRI studies have indicated that lesions of the cerebellum and its connections likely play a primary role in the pathogenesis of tremors in PwMS [26], [33]. Regardless, the presence of lesions in the brainstem, pons, diencephalon, and Guillain-Mollaret triangle (rubro-olivocerebello-rubral loop) can also rarely and independently of the cerebellum, cause severe tremors [26], [34]. Furthermore, previous studies have shown that tremors in PwMS are often reported as intentional with cerebellar lesions and postural with cerebellar and brainstem lesions. Resting phase or Holmes tremors, which occur after damage to the Substantia Nigra pathway leading to the striatum corneum or basal ganglia, are rarely observed [35].

In addition to the location of T2 lesions, the severity of inflammatory responses and lesion load are also probable predictors of the tremor severity observed in PwMS [14], [36], [37]. Previous studies have shown that an increase in lesion load is directly associated with more severe tremors in PwMS and prolongs the recovery time of tremors after relapses [14], [36], [38].

According to previous studies, additional factors may also influence the occurrence of tremors. Disease duration is one of these factors; for example, the study by A. Shalash et al. found that the risk of tremor occurrence increased significantly with increasing disease duration. However, disease duration could not be considered an independent parameter for tremor severity [14].

The EDSS, the primary measurement for measuring disability, is likely directly related to the risk and severity of tremors in PwMS [17], [22]. Gender is another covariate that has been investigated in association with the risk of tremors in PwMS, and although this variable does not have a dramatic correlation with the tremor risk, the study of SH. Alusi et al. indicated that in the MS population, young men are often more prone to tremors than others [22].

The treatment of MS-related tremors remains challenging. Studies have reported the use of buspirone for intention tremors, while other medications such as propranolol, clonazepam, or gabapentin may also provide benefit in some cases [39], [40]. For severe tremors, non-pharmacological approaches like deep brain stimulation or thalamotomy have been explored [41], [42]. Treatment should be individualized based on the patient’s clinical presentation and response.

## 5 Limitations and strengths

To the best of our knowledge, this review represents the first systematic review and *meta*-analysis examining the frequency of tremors among PwMS. However, some limitations of this study must be recognized for a precise interpretation of our findings. The primary limitations include the small number of included studies and their significant heterogeneity. Many studies either did not specify the location of tremor or, when they did, only indicated the affected regions without clarifying whether patients experienced tremor in multiple regions simultaneously. This limited our ability to draw precise conclusions regarding the percentage of tremor in specific anatomical regions. Further research is needed to validate these findings, particularly studies exploring the mechanisms of tremor in PwMS using DTI, fMRI, and neuroimmunological tests to improve our understanding of its pathophysiology. Future studies should also focus on better characterizing the specific types of tremors observed in PwMS, as well as monitoring and controlling for the medications used by patients, to assess their potential influence on tremor severity and progression.

## 6 Conclusion

This preliminary review indicated that tremor is a common complaint among PwMS, with a frequency rate of 22.46 %. These findings underscore the need for focused, supportive, therapeutic, and rehabilitative interventions to effectively manage this widespread issue in PwMS. Further research is needed to validate these findings.

## 7 Declarations

**Ethics approval and consent to participate:** Not applicable.

**Funding:** The authors received no financial support for the research, authorship, or publication of this article.

## CRediT authorship contribution statement

**Saeed Vaheb:** Writing – review & editing, Writing – original draft, Supervision, Investigation, Data curation. **Danial Dehghani Firouzabadi:** Writing – review & editing, Methodology, Investigation, Data curation. **Hamed Ghoshouni:** Methodology, Formal analysis, Data curation. **Mohammad Yazdan Panah:** Writing – review & editing, Software, Methodology, Data curation. **Vahid Shaygannejad:** Writing – review & editing, Supervision, Project administration, Methodology, Investigation. **Omid Mirmosayyeb:** Writing – review & editing, Supervision, Software, Project administration, Data curation, Conceptualization.

## Declaration of competing interest

The authors declare that they have no known competing financial interests or personal relationships that could have appeared to influence the work reported in this paper.
