# Supplementary material for: Frequency of tremor in people with multiple sclerosis: A systematic review and meta-analysis
Source: Clin Park Relat Disord. 2025 Mar 14;12:100315. doi: 10.1016/j.prdoa.2025.100315 (PMC11982949; doi:10.1016/j.prdoa.2025.100315)
Supplement: Supplementary Data 1 [file mmc1.docx]

**Supplementary Material 1**

**Table S1.** The search syntax of each database.

| Database | Results |
| --- | --- |
| PubMed/MEDLINE |  |
| ("Multiple sclerosis" OR "Sclerosis, Multiple" OR "Sclerosis, Disseminated" OR "Disseminated Sclerosis" OR "MS (Multiple Sclerosis)") AND ("Tremor" OR "Intention Tremor" OR "Intention Tremors" OR "Tremor, Intention" OR "Darkness Tremor" OR "Darkness Tremors" OR "Tremor, Darkness" OR "Pill Rolling Tremor" OR "Pill Rolling Tremors" OR "Tremor, Pill Rolling" OR "Fine Tremor" OR "Fine Tremors" OR "Tremor, Fine" OR "Intermittent Tremor" OR "Intermittent Tremors" OR "Tremor, Intermittent" OR "Involuntary Quiver" OR "Involuntary Quivers" OR "Quiver, Involuntary" OR "Massive Tremor" OR "Massive Tremors" OR "Tremor, Massive" OR "Passive Tremor" OR "Passive Tremors" OR "Tremor, Passive" OR "Persistent Tremor" OR "Persistent Tremors" OR "Tremor, Persistent" OR "Resting Tremor" OR "Resting Tremors" OR "Tremor, Resting" OR "Rest Tremor" OR "Rest Tremors" OR "Tremor, Rest" OR "Tremor, Perioral" OR "Perioral Tremor" OR "Perioral Tremors" OR "Tremor, Semirhythmic" OR "Semirhythmic Tremor" OR "Semirhythmic Tremors" OR "Saturnine Tremor" OR "Saturnine Tremors" OR "Tremor, Saturnine" OR "Senile Tremor" OR "Senile Tremors" OR "Tremor, Senile" OR "Static Tremor" OR "Static Tremors" OR "Tremor, Static" OR "Tremor, Limb" OR "Limb Tremor" OR "Limb Tremors" OR "Tremor, Muscle" OR "Muscle Tremor" OR "Muscle Tremors" OR "Tremor, Neonatal" OR "Neonatal Tremor" OR "Neonatal Tremors" OR "Tremor, Nerve" OR "Nerve Tremor" OR "Nerve Tremors" OR "Action Tremor" OR "Action Tremors" OR "Tremor, Action" OR "Coarse Tremor" OR "Coarse Tremors" OR "Tremor, Coarse" OR "Continuous Tremor" OR "Continuous Tremor" OR "Tremor, Continuous") | 633 |
| Scopus |  |
| (TITLE-ABS-KEY("Multiple sclerosis") OR TITLE-ABS-KEY("Sclerosis, Multiple") OR TITLE-ABS-KEY("Sclerosis, Disseminated") OR TITLE-ABS-KEY("Disseminated Sclerosis") OR TITLE-ABS-KEY("MS (Multiple Sclerosis)")) AND (TITLE-ABS-KEY("Tremor") OR TITLE-ABS-KEY("Intention Tremor") OR TITLE-ABS-KEY("Intention Tremors") OR TITLE-ABS-KEY("Tremor, Intention") OR TITLE-ABS-KEY("Darkness Tremor") OR TITLE-ABS-KEY("Darkness Tremors") OR TITLE-ABS-KEY("Tremor, Darkness") OR TITLE-ABS-KEY("Pill Rolling Tremor") OR TITLE-ABS-KEY("Pill Rolling Tremors") OR TITLE-ABS-KEY("Tremor, Pill Rolling") OR TITLE-ABS-KEY("Fine Tremor") OR TITLE-ABS-KEY("Fine Tremors") OR TITLE-ABS-KEY("Tremor, Fine") OR TITLE-ABS-KEY("Intermittent Tremor") OR TITLE-ABS-KEY("Intermittent Tremors") OR TITLE-ABS-KEY("Tremor, Intermittent") OR TITLE-ABS-KEY("Involuntary Quiver") OR TITLE-ABS-KEY("Involuntary Quivers") OR TITLE-ABS-KEY("Quiver, Involuntary") OR TITLE-ABS-KEY("Massive Tremor") OR TITLE-ABS-KEY("Massive Tremors") OR TITLE-ABS-KEY("Tremor, Massive") OR TITLE-ABS-KEY("Passive Tremor") OR TITLE-ABS-KEY("Passive Tremors") OR TITLE-ABS-KEY("Tremor, Passive") OR TITLE-ABS-KEY("Persistent Tremor") OR TITLE-ABS-KEY("Persistent Tremors") OR TITLE-ABS-KEY("Tremor, Persistent") OR TITLE-ABS-KEY("Resting Tremor") OR TITLE-ABS-KEY("Resting Tremors") OR TITLE-ABS-KEY("Tremor, Resting") OR TITLE-ABS-KEY("Rest Tremor") OR TITLE-ABS-KEY("Rest Tremors") OR TITLE-ABS-KEY("Tremor, Rest") OR TITLE-ABS-KEY("Tremor, Perioral") OR TITLE-ABS-KEY("Perioral Tremor") OR TITLE-ABS-KEY("Perioral Tremors") OR TITLE-ABS-KEY("Tremor, Semirhythmic") OR TITLE-ABS-KEY("Semirhythmic Tremor") OR TITLE-ABS-KEY("Semirhythmic Tremors") OR TITLE-ABS-KEY("Saturnine Tremor") OR TITLE-ABS-KEY("Saturnine Tremors") OR TITLE-ABS-KEY("Tremor, Saturnine") OR TITLE-ABS-KEY("Senile Tremor") OR TITLE-ABS-KEY("Senile Tremors") OR TITLE-ABS-KEY("Tremor, Senile") OR TITLE-ABS-KEY("Static Tremor") OR TITLE-ABS-KEY("Static Tremors") OR TITLE-ABS-KEY("Tremor, Static") OR TITLE-ABS-KEY("Tremor, Limb") OR TITLE-ABS-KEY("Limb Tremor") OR TITLE-ABS-KEY("Limb Tremors") OR TITLE-ABS-KEY("Tremor, Muscle") OR TITLE-ABS-KEY("Muscle Tremor") OR TITLE-ABS-KEY("Muscle Tremors") OR TITLE-ABS-KEY("Tremor, Neonatal") OR TITLE-ABS-KEY("Neonatal Tremor") OR TITLE-ABS-KEY("Neonatal Tremors") OR TITLE-ABS-KEY("Tremor, Nerve") OR TITLE-ABS-KEY("Nerve Tremor") OR TITLE-ABS-KEY("Nerve Tremors") OR TITLE-ABS-KEY("Action Tremor") OR TITLE-ABS-KEY("Action Tremors") OR TITLE-ABS-KEY("Tremor, Action") OR TITLE-ABS-KEY("Coarse Tremor") OR TITLE-ABS-KEY("Coarse Tremors") OR TITLE-ABS-KEY("Tremor, Coarse") OR TITLE-ABS-KEY("Continuous Tremor") OR TITLE-ABS-KEY("Continuous Tremor") OR TITLE-ABS-KEY("Tremor, Continuous")) | 1548 |
| Web of Science |  |
| TS=("Multiple sclerosis" OR "Sclerosis, Multiple" OR "Sclerosis, Disseminated" OR "Disseminated Sclerosis" OR "MS (Multiple Sclerosis)") AND (TS=("Tremor" OR "Intention Tremor" OR "Intention Tremors" OR "Tremor, Intention" OR "Darkness Tremor" OR "Darkness Tremors" OR "Tremor, Darkness" OR "Pill Rolling Tremor" OR "Pill Rolling Tremors" OR "Tremor, Pill Rolling" OR "Fine Tremor" OR "Fine Tremors" OR "Tremor, Fine" OR "Intermittent Tremor" OR "Intermittent Tremors" OR "Tremor, Intermittent" OR "Involuntary Quiver" OR "Involuntary Quivers" OR "Quiver, Involuntary" OR "Massive Tremor" OR "Massive Tremors" OR "Tremor, Massive" OR "Passive Tremor" OR "Passive Tremors" OR "Tremor, Passive" OR "Persistent Tremor" OR "Persistent Tremors" OR "Tremor, Persistent" OR "Resting Tremor" OR "Resting Tremors" OR "Tremor, Resting" OR "Rest Tremor" OR "Rest Tremors" OR "Tremor, Rest" OR "Tremor, Perioral" OR "Perioral Tremor" OR "Perioral Tremors" OR "Tremor, Semirhythmic" OR "Semirhythmic Tremor" OR "Semirhythmic Tremors" OR "Saturnine Tremor" OR "Saturnine Tremors" OR "Tremor, Saturnine" OR "Senile Tremor" OR "Senile Tremors" OR "Tremor, Senile" OR "Static Tremor" OR "Static Tremors" OR "Tremor, Static" OR "Tremor, Limb" OR "Limb Tremor" OR "Limb Tremors" OR "Tremor, Muscle" OR "Muscle Tremor" OR "Muscle Tremors" OR "Tremor, Neonatal" OR "Neonatal Tremor" OR "Neonatal Tremors" OR "Tremor, Nerve" OR "Nerve Tremor" OR "Nerve Tremors" OR "Action Tremor" OR "Action Tremors" OR "Tremor, Action" OR "Coarse Tremor" OR "Coarse Tremors" OR "Tremor, Coarse" OR "Continuous Tremor" OR "Continuous Tremor" OR "Tremor, Continuous")) | 707 |
| Embase |  |
| (TI-ABS("Multiple sclerosis") OR TI-ABS("Sclerosis, Multiple") OR TI-ABS("Sclerosis, Disseminated") OR TI-ABS("Disseminated Sclerosis") OR TI-ABS("MS (Multiple Sclerosis)")) AND (TI-ABS("Tremor") OR TI-ABS("Intention Tremor") OR TI-ABS("Intention Tremors") OR TI-ABS("Tremor, Intention") OR TI-ABS("Darkness Tremor") OR TI-ABS("Darkness Tremors") OR TI-ABS("Tremor, Darkness") OR TI-ABS("Pill Rolling Tremor") OR TI-ABS("Pill Rolling Tremors") OR TI-ABS("Tremor, Pill Rolling") OR TI-ABS("Fine Tremor") OR TI-ABS("Fine Tremors") OR TI-ABS("Tremor, Fine") OR TI-ABS("Intermittent Tremor") OR TI-ABS("Intermittent Tremors") OR TI-ABS("Tremor, Intermittent") OR TI-ABS("Involuntary Quiver") OR TI-ABS("Involuntary Quivers") OR TI-ABS("Quiver, Involuntary") OR TI-ABS("Massive Tremor") OR TI-ABS("Massive Tremors") OR TI-ABS("Tremor, Massive") OR TI-ABS("Passive Tremor") OR TI-ABS("Passive Tremors") OR TI-ABS("Tremor, Passive") OR TI-ABS("Persistent Tremor") OR TI-ABS("Persistent Tremors") OR TI-ABS("Tremor, Persistent") OR TI-ABS("Resting Tremor") OR TI-ABS("Resting Tremors") OR TI-ABS("Tremor, Resting") OR TI-ABS("Rest Tremor") OR TI-ABS("Rest Tremors") OR TI-ABS("Tremor, Rest") OR TI-ABS("Tremor, Perioral") OR TI-ABS("Perioral Tremor") OR TI-ABS("Perioral Tremors") OR TI-ABS("Tremor, Semirhythmic") OR TI-ABS("Semirhythmic Tremor") OR TI-ABS("Semirhythmic Tremors") OR TI-ABS("Saturnine Tremor") OR TI-ABS("Saturnine Tremors") OR TI-ABS("Tremor, Saturnine") OR TI-ABS("Senile Tremor") OR TI-ABS("Senile Tremors") OR TI-ABS("Tremor, Senile") OR TI-ABS("Static Tremor") OR TI-ABS("Static Tremors") OR TI-ABS("Tremor, Static") OR TI-ABS("Tremor, Limb") OR TI-ABS("Limb Tremor") OR TI-ABS("Limb Tremors") OR TI-ABS("Tremor, Muscle") OR TI-ABS("Muscle Tremor") OR TI-ABS("Muscle Tremors") OR TI-ABS("Tremor, Neonatal") OR TI-ABS("Neonatal Tremor") OR TI-ABS("Neonatal Tremors") OR TI-ABS("Tremor, Nerve") OR TI-ABS("Nerve Tremor") OR TI-ABS("Nerve Tremors") OR TI-ABS("Action Tremor") OR TI-ABS("Action Tremors") OR TI-ABS("Tremor, Action") OR TI-ABS("Coarse Tremor") OR TI-ABS("Coarse Tremors") OR TI-ABS("Tremor, Coarse") OR TI-ABS("Continuous Tremor") OR TI-ABS("Continuous Tremor") OR TI-ABS("Tremor, Continuous")) | 892 |
